# Volumetric Breast Density Estimation from Full-Field Digital Mammograms: A Validation Study

**DOI:** 10.1371/journal.pone.0085952

**Published:** 2014-01-21

**Authors:** Albert Gubern-Mérida, Michiel Kallenberg, Bram Platel, Ritse M. Mann, Robert Martí, Nico Karssemeijer

**Affiliations:** 1 Department of Computer Architecture and Technology, University of Girona, Girona, Spain; 2 Department of Radiology, Radboud University Medical Center, Nijmegen, The Netherlands; University Medical Centre Utrecht, Netherlands

## Abstract

**Objectives:**

To objectively evaluate automatic volumetric breast density assessment in Full-Field Digital Mammograms (FFDM) using measurements obtained from breast Magnetic Resonance Imaging (MRI).

**Material and Methods:**

A commercially available method for volumetric breast density estimation on FFDM is evaluated by comparing volume estimates obtained from 186 FFDM exams including mediolateral oblique (MLO) and cranial-caudal (CC) views to objective reference standard measurements obtained from MRI.

**Results:**

Volumetric measurements obtained from FFDM show high correlation with MRI data. Pearson’s correlation coefficients of 0.93, 0.97 and 0.85 were obtained for volumetric breast density, breast volume and fibroglandular tissue volume, respectively.

**Conclusions:**

Accurate volumetric breast density assessment is feasible in Full-Field Digital Mammograms and has potential to be used in objective breast cancer risk models and personalized screening.

## Introduction

Breast density has been identified as an important risk factor for developing breast cancer. Studies have reported that the risk of getting breast cancer in women with high breast density is four to six times as large as in women with low breast density [1]–[3]. Additionally, sensitivity of mammography screening is severely impaired in women with high density, since the presence of heterogeneous or extreme dense tissue patterns may obscure suspicious lesions. For this reason, the risk of missing cancers in screening programs increases with density [4]–[6]. Personalization of screening protocols, involving adjunct imaging modalities for women who are currently not adequately screened, has been suggested to circumvent this problem. Such protocols should include risk assessment based on models including family history and breast density biomarkers [7].

To develop such models, it is important to objectively measure breast density. Most studies to date have been performed using subjective visual measurements based on the 4-class Breast Imaging Reporting and Data Systems (BI-RADS) [8], which is used in current clinical practice, or on a visual thresholding technique using dedicated software, such as Cumulus [9]. Both are essentially 2D measurements that determine the area of dense tissue projected in mammograms. Fully automatic methods for area based breast density measurements have been proposed to take subjectivity away [10]–[13]. However, area based measurements do not take the thickness of dense tissue into account. This is a limitation since it is biologically more plausible that breast cancer risk is related to the volume of dense tissue in the breast rather than to its projection [3], [14], [15].

To overcome this limitation, methods for volumetric breast density estimation from mammograms have been proposed [16]–[21]. These methods are based on a physics based model of the X-ray image acquisition process and assume that the breast tissue consists of two types of tissue: fat and parenchyma. By knowing the X-ray attenuation of these tissues, tissue composition at a given pixel can be computed. Initially, researchers have struggled to successfully apply this approach to digitized film mammograms. However, with the introduction of Full-Field Digital Mammograms (FFDM), the development of robust methods and commercial products became possible. Those can be applied to raw (unprocessed) FFDM data, which is made available by all modality manufacturers. Unfortunately, though, raw data is often not archived in clinical practice.

The performance of volumetric breast density estimation methods has been evaluated in several studies. To determine robustness and consistency, comparisons have been made of breast density estimates in the left and right breasts, and in mediolateral oblique (MLO) and cranial-caudal (CC) exposures of the same breast [16], [17]. One would expect to find similar values in CC and MLO views and in regular cases without abnormalities breast density in the left and right breast should be highly correlated. Other studies compared volumetric estimates to BI-RADS density scoring [22], [23]. These previously mentioned validation strategies may not reveal systematic errors, while subjective BI-RADS scorings are coarse and inaccurate by nature and are only useful to determine large errors of the automated methods. Comparison of breast density estimates from FFDM to reference standard measurements obtained from three-dimensional imaging modalities, such as Magnetic Resonance Imaging (MRI) and Computed Tomography (CT), is arguably the most objective and complete validation method [17], [19], [24], [25]. The volume of dense breast tissue can accurately be derived from MR and CT images, as these are 3D acquisitions and no projection is involved. However, quantification of the volume of dense breast tissue is a time consuming task when done by means of manual segmentations because it requires segmentation of 3-dimensional data. For this reason we use computer algorithms to obtain breast density measurements.

In this paper, we evaluate a method for measuring volumetric breast tissue estimates from digital mammograms [17], [19]. We specifically studied the performance of the method for determination of fibroglandular tissue volume, breast volume, and volumetric breast density by comparing its results to volume estimates that were obtained from breast MRI data.

## Materials and Methods

### Dataset

Ethics Statement: According to the Dutch Medical Research Involving Human Subjects Acts (WMO), retrospective studies using only patient records do not require a formal medical ethics review and informed consent is not needed. The need for signed informed consent was waived by the Independent Review Board (IRB). This was confirmed with the local medical ethical committee and can be read at www.ccmo-online.nl. The presented study complies with the Dutch Data Protection Authority requirements on the use of patient data.

In the Radboud University Nijmegen Medical Centre, breast MRI and mammography are used for screening of women with high familial or genetic risk. We included studies for which breast MRI data and FFDM were available with time interval between these exams of less than two months. We obtained 250 MRI volumes and 928 MLO and CC images from FFDM exams from 250 studies (132 different women). Mean time between MRI and FFDM acquisitions was six days. CC views were not available in some cases. All exams were performed between December 2000 and December 2011. The age of the screened women ranged from 24 to 77 years, and was 46.5±11.10 years on average.

The digital mammograms used in the study were acquired on a GE Senographe 2000D or on a GE Senographe DS using standard clinical settings, including the use of an anti-scatter grid. Breast MRI examinations were performed on 1.5 or 3 Tesla scanners (Magnetom Vision, Magnetom Avanto and Magnetom Trio, Siemens) with a dedicated breast coil (CP Breast Array, Siemens). In this study we used pre-contrast T1-weighted MR volumes.

### Breast Density Quantification

In this study, volumetric breast density, breast volume and fibroglandular volume estimates were obtained from FFDM and MRI data. Volumetric breast density refers to the percentage of breast density, computed by dividing the fibroglandular tissue volume by breast volume.

Volumetric estimates from 250 FFDM studies were obtained using Volpara 1.4.3 (Ma?takina, Wellington, New Zealand), which is FDA-approved fully automated software to estimate volumetric breast density. The Volpara method is an extension of the algorithm presented in [17]. In particular, it incorporates a more detailed physics model including scatter components as described in [18], and it uses a more advanced method to determine a reference region of fatty tissue This reference region is used for calibration, and allows computation of fibroglandular tissue thickness at every pixel in the image. Breast volume is determined using a geometric model in which the periphery of the compressed breast is modeled by semi-circular cross sections, using the breast thickness measurement provided by the acquisition system in the image header.

Volumetric measurements from MRI were obtained using a multi-probabilistic atlas-based segmentation method based on [26], [27]. In short, the breast MRI segmentation method initially corrects the bias field and normalizes signal intensities among patients. Secondly, probabilistic atlases, which capture the anatomic variation of the pectoral muscle and chest wall, are used to segment the breast. A probabilistic atlas is a volume that contains the complete spatial distribution of probabilities of voxels to belong to one or more organs [27]. Finally, the fibroglandular tissue is segmented in each breast independently using automatic thresholding. In this work, this method was used to automatically segment breast and fibroglandular tissue in the 250 MRI studies. A radiologist with expertise in breast imaging carefully reviewed all slices of the segmentations and approved 186 (74.4%) MRI studies with segmentations to be suitable for the use as a reference standard for validation of FFDM density measurements. The other 64 (25.6%) studies were excluded from the study. The field of view of 5 of the excluded cases did not entirely cover the breast. In the rest of the excluded cases we observed that the main reason for the MRI segmentation failure was the presence of artifacts or bias field remaining after correction. These signal intensity distortions negatively affected the segmentation process.

### Validation

**Figure 1:**
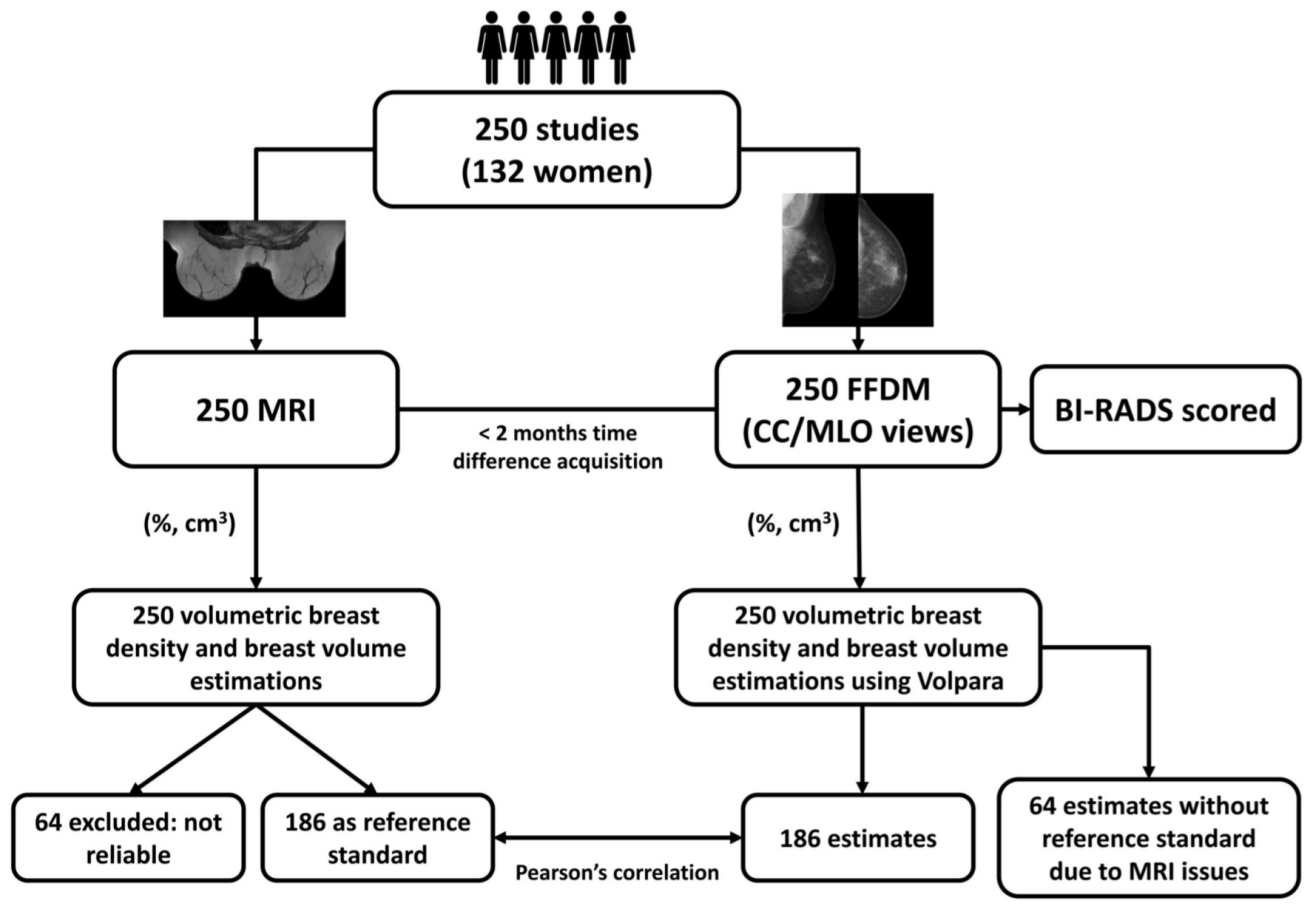
Schematic overview of the validation process.

The validation process is represented in Fig. 1. The Volpara method was validated on 186 FFDM exams including 680 mammographic views. The Pearson’s correlation coefficients between volumetric measures obtained from FFDM and volumetric measures obtained from MRI were calculated per breast and per study. The volumetric estimations per breast from FFDM were averaged over available measures of CC and MLO views for each breast independently. Measures per study were computed by averaging right and left breast volumetric estimates. Because of the log-normal distribution of the data, correlation coefficients were computed after converting the measurements using the natural logarithmic transform [28].

Scatter plots are used to visualize the comparison between breast volumetric estimations. Volpara Density Grade (VDG) thresholds are also shown for volumetric breast density estimates obtained from FFDM. The VDG is a grading system that maps the percent density output of Volpara into four categories similar to the BI-RADS density score. The ranges of the percentage of dense tissue for VDG 1, 2, 3 and 4 are 0 − 4.5%, 4.5 − 7.5%, 7.5 − 15.5% and 15.5% and up, respectively [29].

BI-RADS density scoring (1 to 4) was also performed on the 250 FFDM studies. Each study was classified as (1) fatty, (2) scattered dense, (3) heterogeneously dense or (4) extreme dense by a breast radiologist. Volumetric breast density measurements obtained from FFDMs and MRI, computed per study, were compared to its BI-RADS category provided by the radiologist and the Spearman Ranked correlations were computed for each modality. Finally, to quantify the concordance between VDG and BI-RADS density score, the weighted kappa with quadratic weights coefficient was measured.

## Results

**Figure 2:**
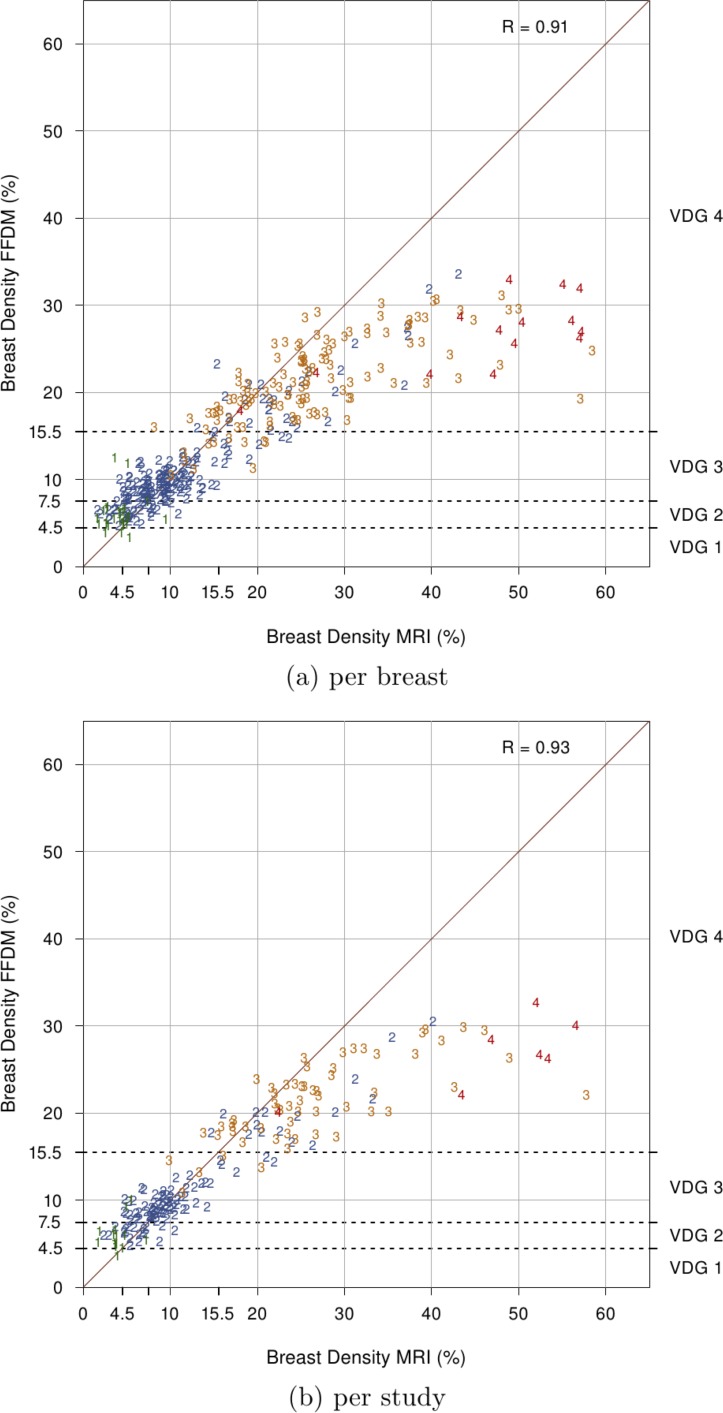
Comparison of percentage of breast density from MRI and FFDMs (a) per breast (n = 353) and (b) per study (n = 186). Each point is labeled with the BI-RADS score. VDG 1, 2, 3 and 4 refer to Volpara Density Grade breast density percentage ranges.

**Figure 3:**
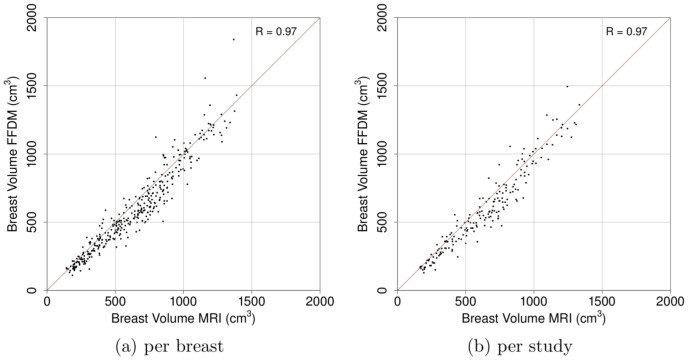
Comparison of breast volume obtained from MRI and FFDMs per (a) breast (n = 353) and (b) per study (n = 186).

**Figure 4:**
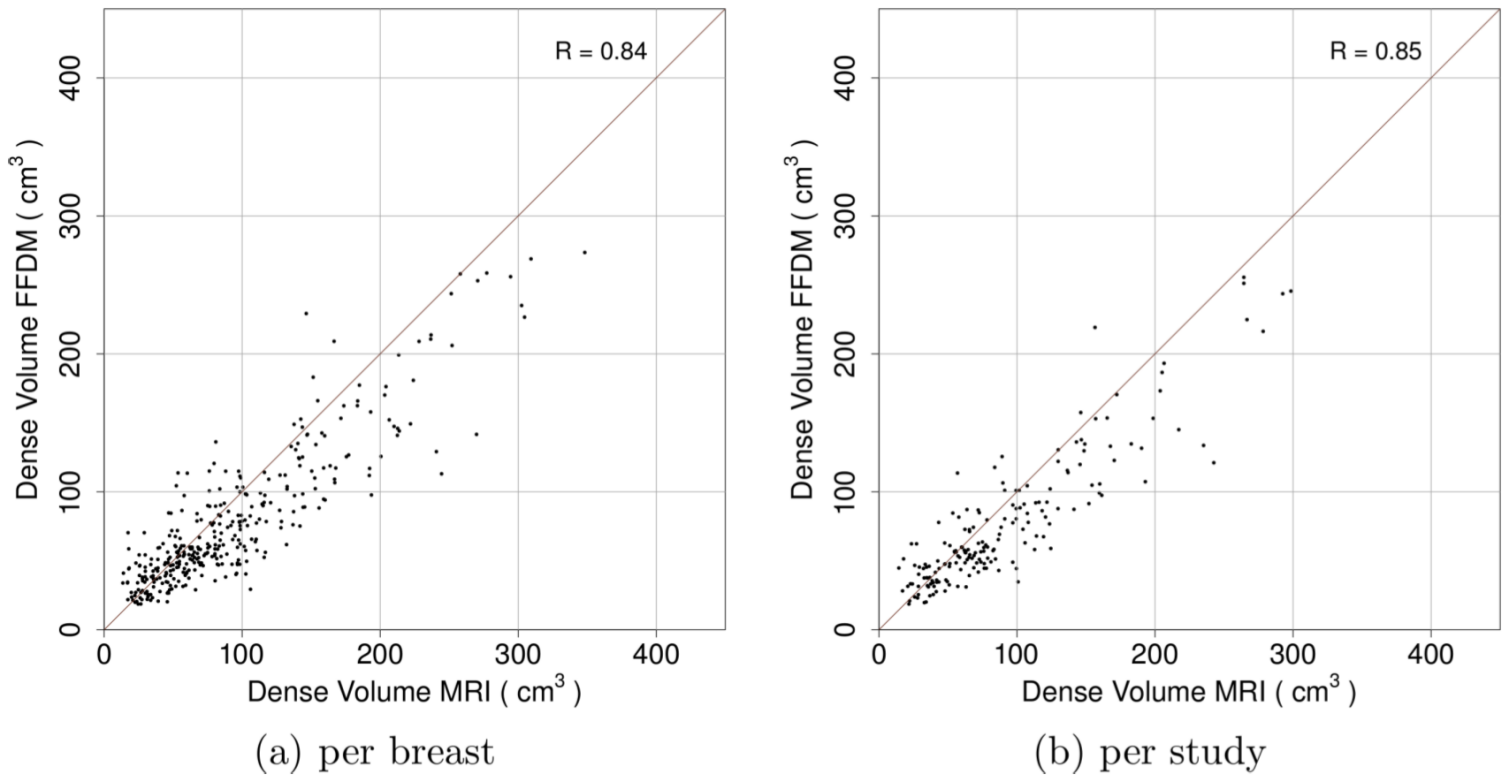
Comparison of fibroglandular tissue volume obtained from MRI and FFDMs (a) per breast (n = 353) and (b) per study (n = 186).

Table 1 summarizes the results obtained in this validation study. Figure 2 shows the relation between percentage of volumetric breast density from mammograms and MRI data per breast (a) and per study (b). Correlations per breast and per study are 0.91 and 0.93, respectively. Figure 3 shows the relation between breast volume estimates from mammograms and MRI data. Per breast (a) and per study (b) correlations are 0.97 and 0.97, respectively. Additionally, Fig. 4 shows the relation between fibroglandular tissue volume estimates from mammograms and MRI data. Correlation per breast (a) is 0.84 and correlation per study (b) is 0.85.

**Table 1:** Summary of the dataset and the results obtained in this study.

Number of studies	186	
Number of mammographic views	680	
Number of breasts	353	
	**FFDM (Median (IQR))**	**MRI (Median (IQR))**
Volumetric breast density (%)	11.90 (12.86)	13.55 (17.15)
Breast volume (cm^3^)	551.95 (405.32)	643.16 (439.56)
Fibroglandular tissue volume (cm^3^)	60.45 (50.36)	76.27 (72.20)
	**Per breast**	**Per study**
Volumetric breast density correlation		
- FFDM - MRI	0.91*	0.93*
- FFDM - BI-RADS	–	0.78+
- MRI - BI-RADS	–	0.77+
- VDG - BI-RADS	–	0.40−
Breast volume correlation		
- FFDM - MRI	0.97*	0.97*
Fibroglandular tissue volume correlation		
- FFDM - MRI	0.84*	0.85*

IQR = inter-quartile range,

* = Pearson correlation coefficient,

+ = Spearman Ranked correlation coefficient,

− = weighted kappa with quadratic weights coefficient.

Overall, high correlation between FFDM and MRI measurements iss observed. However, results indicate that Volpara tends to underestimate breast density in dense breasts compared to MRI. Correlation drops for volumetric breast density measurements classified within the VDG 4 range.

**Figure 5:**
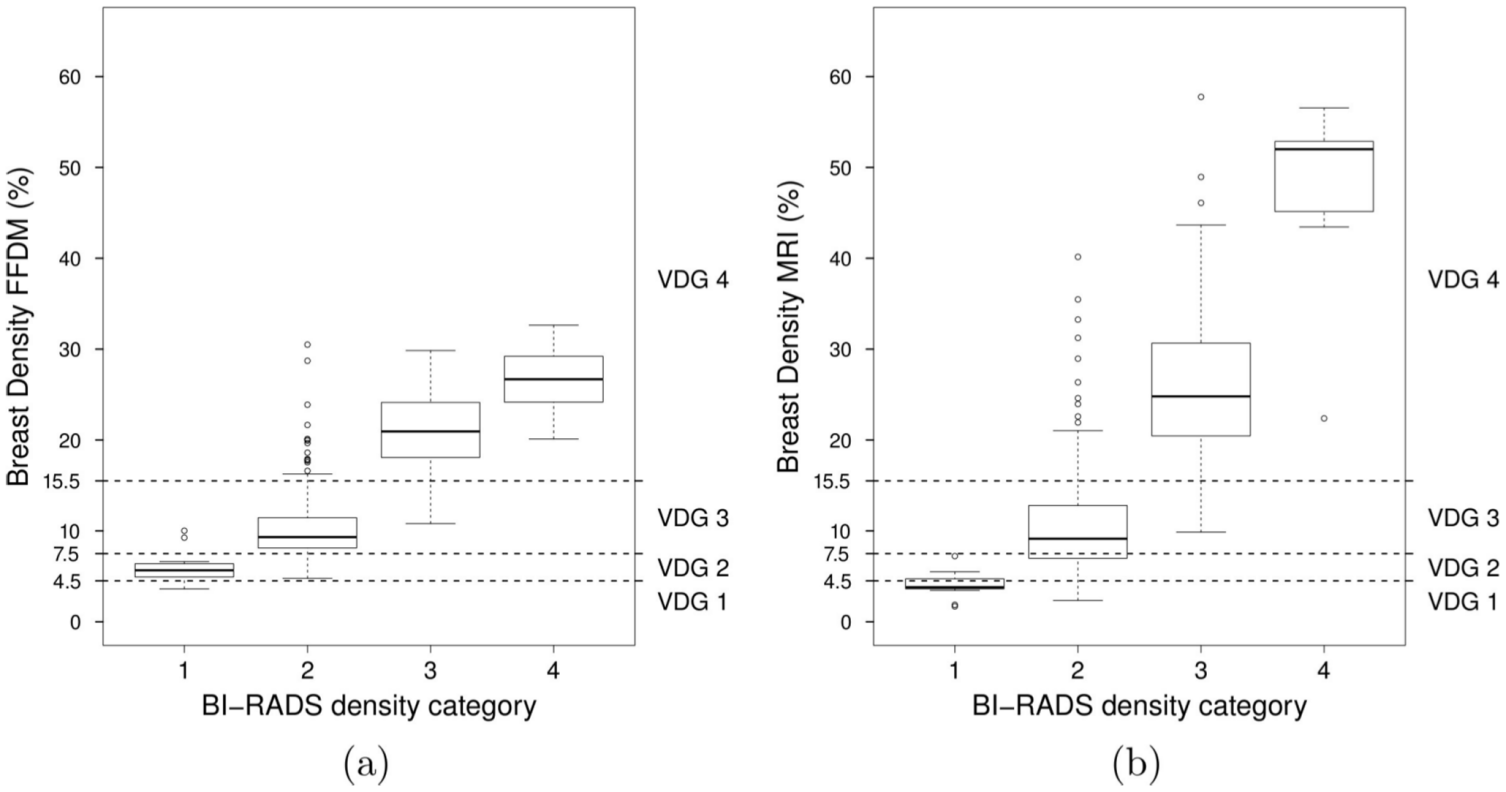
Association between volumetric breast density estimates per study and BI-RADS category.

**Figure 6:**
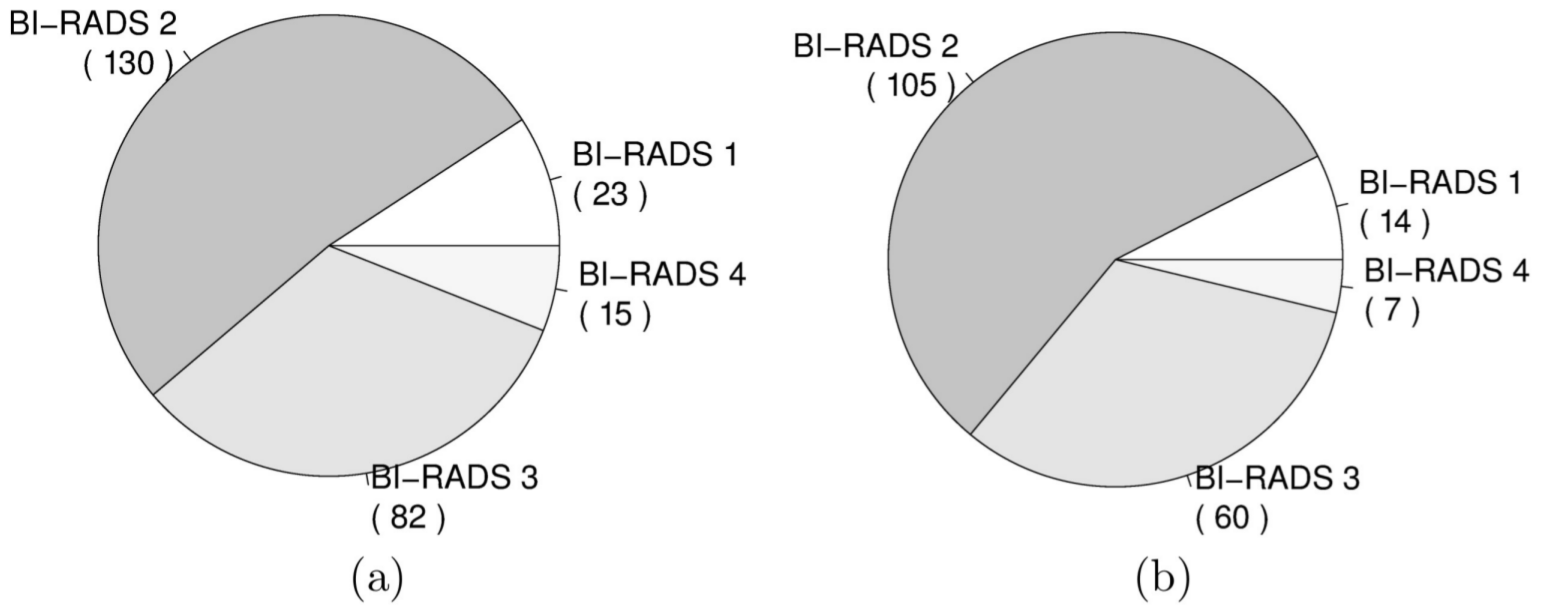
Frequency of studies scored with BI-RADS categories 1, 2, 3 and 4 for (a) the complete dataset (n = 250) and (b) for the cases of the dataset with reference standard estimates (n = 186).

Furthermore, Fig. 5 shows the association between volumetric breast density estimates and BI-RADS category. The estimates are obtained from FFDMs on Fig. 5(a), and obtained from MRI on Fig. 5(b). Spearman Rank correlation coefficients are 0.79 and 0.78 for FFDM and MRI, respectively. The reported correlations are not statistically significantly different (p-value = 0.71, two-tailed z-test). Following the trend observed before, volumetric breast density estimates are larger when obtained from MRI than when computed on FFDMs. The median estimates obtained with Volpara range from 5.66%, in the lowest BI-RADS category, to 26.69%, in the top category. Median estimates obtained from MRI data range from 3.80% to 52.00%. Figure 6 shows the number of studies scored with BI-RADS categories 1, 2, 3 and 4 for (a) the initial dataset and for (b) the dataset after excluding studies with poor MR segmentations. Finally, Table 2 shows the confusion matrix for the VDG using the Volpara method versus BI-RADS density score given by the breast radiologist. The weighted kappa with quadratic weights statistic was 0.40.

**Table 2:** Volpara Density Grade (VDG) versus BI-RADS density score from a breast radiologist.

		BI-RADS				
		1	2	3	4	
	**1**	2	0	0	0	2
**Volpara (VDG)**	**2**	10	19	0	0	29
	**3**	2	70	5	0	77
	**4**	0	16	55	7	78
		14	105	60	7	186

## Discussion

In this study we have presented a validation of Volpara 1.4.3 (Mātakina, Wellington, New Zealand), which is a commercially available method for assessing volumetric breast density on FFDM. Volpara has been evaluated on 186 studies including 680 mammographic views of 353 breasts in total. Volumetric estimates obtained from FFDM have been compared to objective reference standard measures computed from MRI. Volumetric breast density and breast tissue volume values obtained with Volpara present high correlation when compared to MRI measurements. To date, this is the largest validation study that compares volumetric breast density estimates from FFDM to reference standard measurements obtained from MRI, a 3D imaging modality.

In previous work, Wang et al. [25] used a dataset of 123 patients and also compared volumetric measurements obtained from FFDM to estimates obtained from MRI. Correlations for breast volume, fibroglandular tissue volume and volumetric breast density were 0.94, 0.62 and 0.71, respectively. We found higher correlation values than the ones reported in their work (R = 0.97, R = 0.85 and R = 0.93 for breast volume, fibroglandular tissue volume and volumetric breast density, respectively). Van Engeland et al. [17] also compared density estimates from FFDM to estimates from MRI in a small study including 22 patients, but only reported correlation between fibroglandular tissue volume from mammograms and from MRI data. The correlation was 0.97. In our study we found a lower correlation between fibroglandular tissue volume from FFDM and from MRI (R = 0.84). In previous studies, Volpara was also compared to semi-automatic area-based density measurements. High correlation between the volumetric breast density obtained with Volpara and area-based percentage density using Cumulus was found (R = 0.85) [23]. Care should be taken when comparing the correlation coefficients obtained in this work to the values reported in similar studies; these similar studies were performed on different datasets. In our study, the dataset was mostly composed of pre-menopausal women participating in a high-risk screening program. In this dataset, a different distribution of breast density may be expected when compared to breast density distributions of other datasets, since there are many factors that influence breast density (such as age and use of hormone replacement therapy). On the other hand, we may assume that the appearance of fibroglandular tissue itself in our study group is similar to that in other studies, since there is no evidence that breast density patterns in women in a high risk population differ from those in the general population.

Compared to volumetric measurements obtained from MRI, results show that Volpara tends to underestimate breast density in very dense breasts. This effect has been also observed in other methods for volumetric breast density estimation [17], [30]. Like Volpara, these methods are also based on a physics-based image model and, to predict fibroglandular tissue thickness, use a set of pixels of the breast that belong to fatty tissue as an internal reference. The selection of the internal reference is more complex in dense breasts than in fatty breasts, which affects the calibration of fatty tissue attenuation and leads to breast density underestimation. However, the breast density underestimation in dense cases does not seem to affect the final VDG categorization. We observed that the cases with the largest negative difference between estimates from FFDM and MRI obtained a volumetric breast density estimate from FFDM greater than 15% and were classified as VDG 4.

Compared to BI-RADS density scores given by a breast radiologist, a clear association is observed, but low agreement between VDG scores and BI-RADS density scores was found (weighted kappa with quadratic weights coefficient = 0.40). In general, VDG scores tend to be higher than the BI-RADS density scores. For instance, 70 studies that were scored with BI-RADS 2 obtained a VDG score of 3. The same trend was observed on 55 studies that were scored with BI-RADS 3, which obtained a VDG of 4. One should note that the VDG thresholds were set based on a US radiologist’s assessment of BI-RADS density. The low agreement and the perceived overestimation might be caused by the fact that the BI-RADS scoring in this work was done by an European radiologist. BI-RADS density grades have been suggested to be underestimated according to EU standards when compared to US radiologist [31]. However, further research is still required to investigate this effect as only a single radiologist participated in the presented study.

Regarding the validation process, it was a limitation of our study that we had to exclude cases without reliable breast MRI fibroglandular tissue segmentation. However, we do not think this influenced our results because the causes for rejecting MRI cases were mostly not related to breast composition. Rejected cases were distributed evenly for the BI-RADS categories 1, 2 and 3. A higher percentage of rejected cases was observed on BI-RADS category 4 (8 of 15). This fact is explained by the difficulty of automatically segmenting fibroglandular tissue in breasts with high density in MRI. One could think that the exclusion of these BI-RADS category 4 cases increases the correlation coefficients between FFDM and MRI measurements. However, these rejected cases had minor influence on the complete dataset (3% of the total number of studies).

In conclusion, our study shows that it is feasible to obtain accurate measurements of absolute and relative volumes of dense breast tissue from full field digital mammograms. Availability of such measurements is crucial for the development of objective breast cancer risk models and may be used in the development of personalized screening protocols.
